# Preparation and Characterization of Enzyme Compartments in UV-Cured Polyurethane-Based Materials and Their Application in Enzymatic Reactions

**DOI:** 10.3389/fmicb.2017.02111

**Published:** 2017-11-09

**Authors:** Diana Uhrich, Jan von Langermann

**Affiliations:** Biocatalysis Group, Institute of Chemistry, University of Rostock, Rostock, Germany

**Keywords:** biocatalysis, immobilization, alcohol dehydrogenase, inclusion, organic solvent

## Abstract

The preparation and characterization of UV-cured polyurethane-based materials for the mild inclusion immobilization of enzymes was investigated. Full curing of the polymer precursor/enzyme solution mixture was realized by a short irradiation with UV-light at ambient temperatures. The included aqueous enzyme solution remains highly dispersed in the polymer material with an even size distribution throughout the polymer material. The presented concept provides stable enzyme compartments which were applied for an alcohol dehydrogenase-catalyzed reduction reaction in organic solvents. Cofactor regeneration was achieved by a substrate-coupled approach via 2-propanol or an enzyme-coupled approach by a glucose dehydrogenase. This reaction concept can also be used for a simultaneous application of contrary biocatalytic reaction conditions within an enzymatic cascade reaction. Independent polymer-based reaction compartments were provided for two incompatible enzymatic reaction systems (alcohol dehydrogenase and hydroxynitrile lyase), while the relevant reactants diffuse between the applied compartments.

## Introduction

Enzymes are highly versatile biocatalysts that catalyze a vast variety of reactions under typically mild reaction conditions (Faber, 2004; Tao and Kazlauskas, 2011). In their natural environment these enzymes are also often localized within cellular or subcellular domains (microenvironment compartmentalization), which provides optimal conditions for the respective reaction, e.g., pH and availability of (co-)substrates, cofactors, etc. (Ovádi and Saks, 2004; Zecchin et al., 2015). This spatial organization facilitates also an easy transfer of metabolites from one catalytic domain to the next, yielding an effective assembly line with increased overall output of the desired product. For example, eukaryotic cells are based on a complex of membranes and other compartments, which are specialized for the required biological functions (Huh et al., 2003; Barabási and Oltvai, 2004). These compartments are not necessarily permanent, as found in the metabolic cycle of yeast, which features temporal compartments (Tu et al., 2005). In general, such permanent and temporal compartments allow within a complex orchestra of reaction pathways biological systems to perform anabolic and catabolic processes in a coordinated approach with an effective usage of resources. In contrast, the use of enzymes for synthetic purposes frequently requires non-physiological reaction conditions, e.g., use of an organic solvent or extreme pH, which are not compatible with classical natural compartments. For this purpose tailor-made artificial compartments are required that provide sufficient applicability under non-conventional conditions. The enzyme remains similarly to their natural analogs segregated from the external solution within the compartment. However, such artificial concepts require (a) a careful control of the preparation process to facilitate catalytically active enzymes in the non-natural compartments, (b) sufficient chemical and mechanical stability at the chosen non-conventional reaction conditions, and (c) a relatively low diffusion limitation of the reactants through the respective support material to avoid bottlenecks in the synthesis process. Currently a rather limited selection of synthetic compartmentalization options is available for such purposes, which include predominantly the application of sol-gels, silicone-based “static emulsions” and less frequently the use of full solution inclusions/cell mimicry, e.g., in polysiloxane and polymersomes (Pierre, 2004; Buthe et al., 2005; Ansorge-Schumacher, 2007; Wu et al., 2011; Zhang et al., 2011; Patterson et al., 2014; Klermund et al., 2017). Unfortunately sol gel-concepts include relatively harsh conditions, e.g., the use of alkylsilanes and at least one drying processes, which alters the apparent matrix and thus often affects the compartmented enzyme negatively (Cabirol et al., 2006). An inclusion of an entire aqueous phase in a compartment is in theory preferred since the enzyme remains in the aqueous solution, but requires often complex and time-consuming preparation procedures.

**FIGURE 1:**
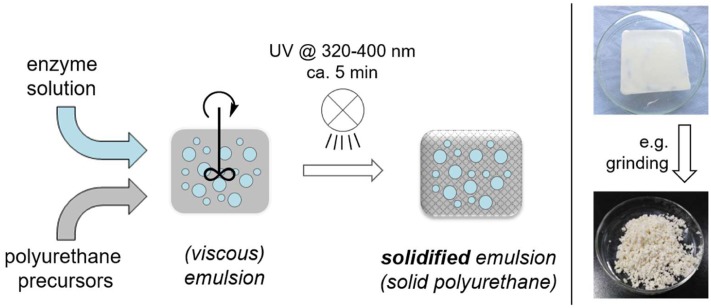
General preparation of UV-cured enzyme compartments in polyurethane.

In this study, we present a simplified and very fast approach to enzyme compartments via UV-curable polymer preparations and their use for synthetic purposes (Norland_Products, 2017). Herein a hydrophobic polyurethane precursor is mixed with an aqueous enzyme solution forming a viscous emulsion, which is then solidified by UV-irradiation within ca. 5 min at ambient temperature (Carlborg et al., 2011). In the resulting solid material the original aqueous enzyme solution remains as small droplets dispersed in the polymer matrix (**Figure 1**). The obtained solid material is eventually cut or grinded into the desired size to minimize potential reactant diffusion limitations through the polymer network. The alcohol dehydrogenase-catalyzed reduction of prochiral ketones to the respective chiral alcohols was chosen as a model reaction for applicability.

Moreover, enzymes usually operate at relatively similar reaction conditions and a concurrent application of more than one isolated enzyme in the same reaction vessel is obvious. Consequently numerous process concepts were reported for synthetic purposes (Schrittwieser et al., 2011; Sánchez-Moreno et al., 2012; Guterl and Sieber, 2013; Oberleitner et al., 2013; Oroz-Guinea and García-Junceda, 2013; Riva and Fessner, 2014; Muschiol et al., 2015). In general, four different designs of enzymatic cascade (tandem) reactions are possible: linear, parallel, orthogonal, and cyclic cascade (Ricca et al., 2011; Simon et al., 2014). Examples include, aside the above mentioned multi-enzymatic approaches, numerous variations of organo- or (transition) metal catalysts in combination with biocatalysts (Gelman et al., 2002; Köhler and Turner, 2014). The use of such reaction cascades typically facilitates three main advantages: (a) no need to isolate intermediates, which significantly reduces efforts in downstream processing and improves overall yield, (b) avoiding inhibiting or toxic concentrations of intermediates, and (c) overcoming thermodynamically unfavored reaction equilibria by pushing the reaction to the product side, which again improves overall yield of the process (Ricca et al., 2011; Abu and Woodley, 2015). On the other hand, the presence of multiple reagents in one solution may cause unwanted secondary (catalytic) side reactions and thus a careful selection and control of the reaction process is still required. Fortunately, compartmentation of reaction zones can be used to (partly) overcome certain limitations of such (bio)catalytic cascade reactions since optimal reaction conditions for each (bio)catalytic reaction system can be selected. Thus, the presented polyurethane-based material was used for the preparation of two different, non-compatible aqueous conditions and subsequently used in a biocatalytic cascade reaction. As a model reaction a combination of two very contrary reaction systems was chosen, the alcohol dehydrogenase from *Lactobacillus kefir*, *Lk*ADH and hydroxynitrile lyase from *Manihot esculenta*, *Me*HNL.

## Materials and Methods

### General

Acetophenone, aliphatic ketones, 2-propanol, triethanolamine (TEA), citric acid, dipotassium phosphate, and methyl tert-butyl ether (MTBE) were obtained from Sigma-Aldrich, Seelze, Germany. Magnesium chloride, potassium dihydrogen phosphate, mandelonitrile, glucose, and concentrated hydrochloric acid were purchased from Merck, Darmstadt, Germany. Sodium cyanide was received from Fluka Chemika AG, Buchs, Switzerland. In addition, mandelonitrile was stored at -18°C to minimize decomposition to benzaldehyde and hydrogen cyanide. Sodium hydroxide was purchased from VWR International, Darmstadt, Germany and calcium carbonate was obtained from Fisher Scientific, Loughborough, United Kingdom. NOA81 was obtained from Thorlabs, Dachau, Germany. All chemicals were obtained in highest available purity and used as received. The hydroxynitrile lyase from *Manihot esculenta* was a gift from Jülich Fine Chemicals (now Codexis). Alcohol dehydrogenase from *Lactobacillus kefir* (*Lk*ADH) and glucose dehydrogenase (GDH) were purchased from evocatal, Monheim, Germany. Deionized water was produced with an Ultra Clear Reinstwassersystem by SG Water (now Evoqua, Guenzburg, Germany) and used throughout this study.

### Analytical Methods

Enzyme activity was measured with spectrophotometer Specord 50 from Analytik Jena, Jena, Germany. Conversion and enantiomeric excess’ of all reactions were measured by gas chromatography with a Trace 1310 gas chromatograph (from Thermo Scientific, Dreieich, Germany) with a 1300 flame ionization detector equipped with a Chirasil-Dex-CB-column (25 m × 0.25 mm × 0.25 μm). Carrier gas helium (purity: 99.999%) with a flow rate of 1.7 mL/min was used throughout this study. Temperatures of the injector and detector were set to 250°C. Temperature program: 60°C for 4 min, followed by a heating rate of 5 K/min to 140°C. NMR spectra were recorded in CDCl3 with a Bruker Avance 250 MHz (Rheinstetten, Germany).

### Enzyme Assays

Alcohol dehydrogenase activity was determined by monitoring the consumption of NADPH at 340 nm over 3 min at 30°C. Assay conditions: A standard reaction solution of 1 mL contained 970 μL of 11 mM acetophenone with 1 mM MgCl_2_ in 50 mM TEA buffer pH 7, 20 μL 12.5 mM NADPH and 10 μL enzyme sample. Specific activity was 43.3⋅Umg^-1^. Hydroxynitrile lyase activity was measured spectrophotometrically by monitoring the release of benzaldehyde from racemic benzaldehyde cyanohydrin (mandelonitrile) over 3 min at 280 nm. Assay conditions: 25°C, 900 μL 50 mM citrate buffer pH 5, 50 mL stock solution (100 mM) of rac-mandelonitrile (HNL) in 10 mM citric acid solution and 50 μL enzyme sample solution. Volumetric activity was 96 U⋅mL^-1^. All measurements were executed in triplicate (error bars indicate standard deviation), and the spontaneous reaction subtracted. One unit of enzyme activity is defined as the conversion of 1 μmol substrate per minute under assay conditions. Extinction coefficients: ADH-assay: ε (340 nm, NADPH) = 6220 L⋅mol^-1^⋅cm^-1^: HNL-assay: ε (280 nm, benzaldehyde) = 1352 L⋅mol^-1^⋅cm^-1^.

### Compartmentalization of Enzymes

For a typical immobilization 1 g NOA 81 were manually emulsified with 400 μL of 5 mg/mL enzyme solution (containing also 1 mM cofactor in 50 mM phosphate or TEA buffer; in case of GDH: 100 mM glucose and if required 20 mg CaCO_3_) for 1 min, spread out to thickness of 1 mm and subsequently irradiated with UV-light (366 nm) for 5 min (no stirring). Irradiation was executed with a NU-4 UV Hand Lamp (4 W) by Herolab GmbH Laborgeräte (Wiesloch, Germany) at a distance of 12 cm in a closed container (23 cm × 18 cm × 11 cm). The obtained solid particles were cut into small pieces (2–5 mm outer length) by a Cloer electric mill (200 W) (Cloer Elektrogeräte, Arnsberg, Germany) to facilitate a fast exchange of reactants.

### Microscopy

The size distribution was directly measured from microscopic images of freshly cut polymeric materials. The images covered each an area of ca. 1.1 mm^2^. SEM analysis (scanning electron microscopy) was performed using the Supra-25 from Carl Zeiss AG, Jena, Germany. The Microscope was equipped with a field emission gun and InLens, SE, 4QBSD, EDX detectors. Before measurement, the samples were positioned on a wafer and coated with palladium/platinum layer (approximately 5 nm). The analysis was run at 10 kV. Particle size was estimated using the Alicona MeX stereoscopic image analysis software.

### Hydrogen Cyanide Preparation

All reactions including or forming hydrogen cyanide were performed in a well ventilated fume hood for self-protection. An electrochemical hydrogen cyanide detector Micro III G203, from GfG-Gesellschaft für Gerätebau, Dortmund, Germany was used throughout this study for continuous monitoring. The required amount of sodium cyanide was dissolved in ca. 10-fold amount of deionized water and cooled to 5°C. Afterward 5 M HCl was slowly added via a dropping funnel to the cyanide solution, while the internal temperature was kept below 10°C to avoid evaporation of hydrogen cyanide. After completion of the reaction the resulting solution was extracted twice with MTBE. The HCN-solution in MTBE was used for the compartmented biocatalytic cascade reaction without any further purification (see also below).

### General Reaction Procedure

In a typical experiment the required amounts of acetophenone and 2-propanol were dissolved in 5 mL MTBE. Afterward the compartmented enzyme (see above) was added and the resulting suspension shaken horizontally at 30°C with an IKA HS 260c shaker at 180 rpm (IKA-Werke, Staufen, Germany). In case of a two-enzyme cascade reaction the above mentioned HCN-solution (in MTBE) was used instead. The compartmented *Me*HNL was also added to the solution and the resulting mixture stirred at room temperature (21°C) in a fume hood. Samples for reaction control and determination of enantiomeric excess (gas chromatography) were directly taken from the outer solvent. The measurements were executed in triplicate and the mean value presented (error bars indicate standard deviation).

## Results and Discussion

### Characterization of Aqueous Compartments

**FIGURE 2:**
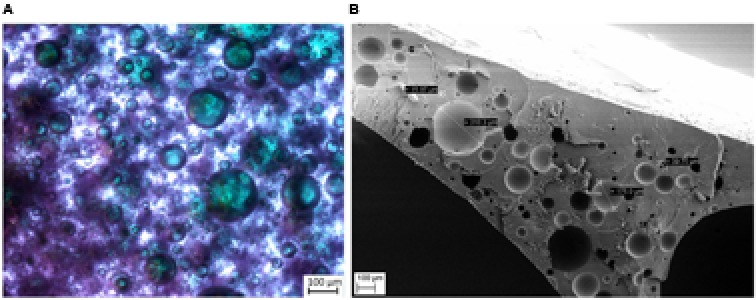
Exemplary microscopic images of aqueous inclusions in a polyurethane matrix. **(A)** Light microscope image: spherical inclusions (highlighted blue/green by an aqueous solution of bromophenol blue) in the polyurethane matrix. **(B)** SEM image of a polyurethane cross section.

The preparation of the polyurethane-based compartments is based on a simple two-step process. First, the hydrophobic polyurethane precursor material (NOA 81 by Norland Products) is vigorously mixed with an aqueous solution, which contains all required enzymes, cofactors, co-substrates, and buffer salts (Norland_Products, 2017). This results in a viscous water-in-oil-emulsion with small independent droplets of the original aqueous phase. Due to the high viscosity of the precursor material an additional use of an emulsifier is not required. Subsequently curing of this mixture with UV-light eventually initiates polymerization of the polyurethane precursors, forming a solid, rubber-like material. The aqueous inclusions are not directly affected by polymerization and remain physically fixed as full medium inclusion in the polymer matrix. This phase includes all dissolved or otherwise suspended compounds in the aqueous phase, while polyurethane forms a physical barrier/membrane around it, which prevents any further coalescence of the aqueous droplets into a larger bulk phase. The dispersed aqueous inclusions appear randomly positioned within the solid polymer material. The distribution of the aqueous inclusions was visualized by dyeing the aqueous domains with bromophenol blue, while the polymer itself remains transparent (**Figure 2A**). Air inclusions, originating from the preparation process, are occasionally found as ‘empty’ pockets in the polymer. REM-images of freshly cut preparations show clearly the highly spherical nature of these aqueous inclusions (**Figure 2B**). This behavior originates back to the uncured emulsion, which minimize interfacial tension by a low surface to volume ratio in such spherical form.

Up to 37.5 wt% of an aqueous phase can be incorporated into the final polymer preparation without any noticeable difference to polymer preparation without any included aqueous phases. Higher amounts of water causes insufficient curing to gel-like materials, which are not applicable as enzyme compartments. Also high protein concentrations do not affect curing of the emulsion. Up to 20 mg/mL of a model protein (bovine serum albumin) were incorporated without interfering with the curing process, which indicates the separation of the aqueous domains from the curing process within the polymer network. Noticeable leaching of protein from polyurethane compartments was not found, e.g., for *Lk*ADH.

**FIGURE 3:**
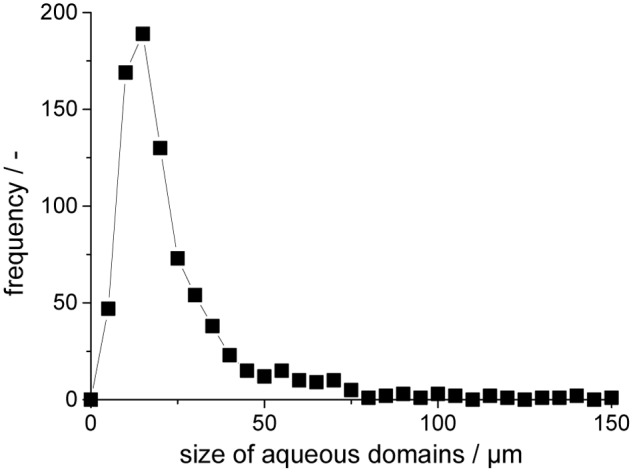
Size distribution of aqueous inclusions in the polyurethane preparation.

The actual size distribution of the aqueous inclusions is directly influenced by the stirring of the precursor/water-emulsion before UV-curing locks its size and position. Within this study the majority of inclusions are in a range of 5–75 μm with occasionally larger inclusions of >100 μm (**Figure 3**). Larger inclusions obviously hold more volume of an aqueous phase and thus more catalytic activity, but smaller domains provide a very high surface area for a fast exchange of reactants via its water-solid polymer-interface.

The obtained polymer preparations are meant to be used in organic solvents to ensure high solubilities for hydrophobic reactants. Therefore water-loaded polyurethane preparations were tested against various organic solvents with different polarities. All investigated organic solvents do not cause disintegration of the polymer matrix, but swelling occurs for a number of solvents. Strong swelling was found using DMSO and THF, moderate swelling with toluene and diethyl ether and no swelling was observed with MTBE, *n*-butanol, acetone, and ethanol. In addition, acidic solutions are easily tolerated by these compartment preparations, but strong bases will hydrolyze the polyurethane material very easily.

### Alcohol Dehydrogenase-Catalyzed Reaction

**FIGURE 4:**
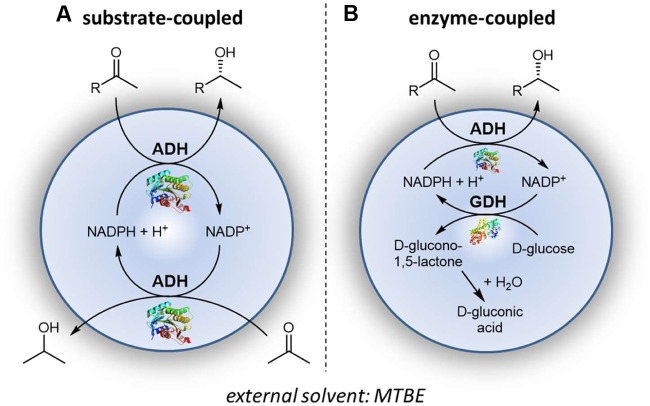
Compartmentalized *Lk*ADH-catalyzed reaction with **(A)** substrate coupled and **(B)** enzyme coupled cofactor regeneration.

The applicability of polyurethane-based compartments was investigated in detail for the reduction of prochiral ketones with the alcohol dehydrogenase from *Lactobacillus kefir*, *Lk*ADH. This includes cofactor regeneration by two commonly used regeneration concepts, using (a) co-substrate 2-propanol in a substrate-coupled approach (**Figure 4A**) and (b) D-glucose in an enzyme-coupled approach using glucose dehydrogenase (**Figure 4B**) (Eckstein et al., 2004; Kara et al., 2014).

In both cases cofactor regeneration takes place directly within the polyurethane compartments, consistent with the compartmentation concept. Simultaneously the final product (chiral alcohol) enriches throughout the reaction in the external solvent phase. In case of the substrate-coupled approach co-product acetone accumulates in the external solvent phase as well and can be removed from there without interfering with the enzyme, cofactor or water phase in general, e.g., by evaporation. A noteworthy exception is the use of glucose dehydrogenase for cofactor regeneration since D-glucose, D-glucono-1,5-lactone, and D-gluconic acid remain fully compartmentalized in the polyurethane preparation. The final co-product D-gluconic acid can be easily captured in the buffered solution or at co-compartmentalized calcium carbonate. In this case, only the desired chiral alcohol enriches in the external solvent phase.

**FIGURE 5:**
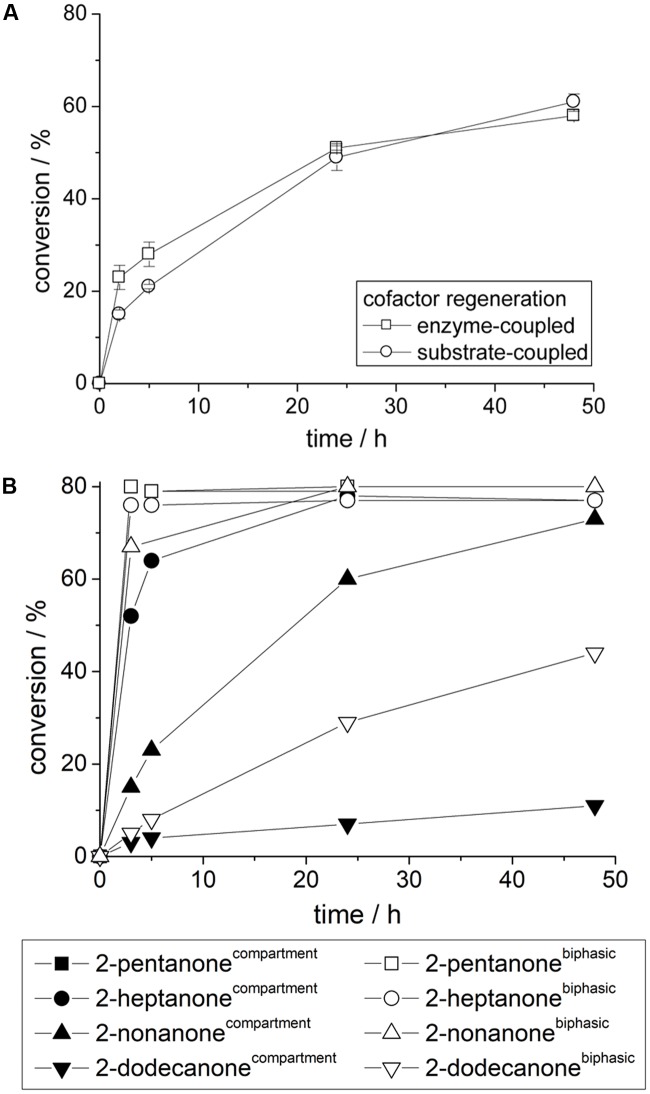
**(A)** Reaction of compartmented *Lk*ADH with enzyme-coupled and substrate-coupled cofactor regeneration. **(B)** Comparison of compartmented *Lk*ADH and a classical biphasic system for the conversion of various prochiral aliphatic ketones; Reaction conditions: Compartmented *Lk*ADH: 10 mM substrate, 100 mM 2-propanol in 5 mL MTBE (external solvent); biphasic reaction system: 10 mM substrate, 100 mM 2-propanol in 1 mL MTBE and 4 mL 50 mM phosphate buffer pH 7.5 (incl. identical amount of dissolved *Lk*ADH).

Regardless of the applied cofactor regeneration concept similar reaction velocities were obtained with compartmented alcohol dehydrogenase from *Lactobacillus kefir* for the conversion of acetophenone (**Figure 5A**). The shown reaction velocity can be further improved by higher alcohol dehydrogenase concentrations (data not shown). This indicates again that *Lk*ADH and the applied cofactor regeneration reaction are generally not affected by the polymerization procedure and fully compartmentalized. After curing the transport of reactants is solely achieved by diffusion through the cured polymer network into the aqueous inclusion. Swelling of polyurethane (see also above) by organic solvents also contributes to the transport phenomena, but seem to improve transport in general. Similarly other prochiral ketones can be easily converted by compartmentalized alcohol dehydrogenase with high enantioselectivities (**Figure 5B**). Herein especially smaller and more polar substrates are reduced very fast by compartmented *Lk*ADH, e.g., 80% equilibrium conversion for 2-pentanone was obtained within a few hours, while longer reaction times are required for 2-nonanone and even larger substrates. In comparison, a classical biphasic reaction system yields similar identical conversions (Eckstein et al., 2006), however, larger substrates are converted faster in comparison to compartmentalized *Lk*ADH. This effect seems to be a combination of two main effects. First, diffusion limitation of large, unpolar reactants, while smaller, polar molecules such as 2-propanol and (*R*)-2-pentanol diffuse rather fast through the polyurethane membrane. Second, unpolar substrates such as 2-dodecanone partition into the external organic solvent and also unpolar polymer material, which results in a lower substrate concentration in the compartmented aqueous domain, e.g., even below the respective *K*_M_-value, which yields lower apparent activities for such large unpolar substrates.

In addition, the stability of the obtained enzyme compartments was investigated to ensure sufficient applicability also after prolonged storage times. Reaction time courses after given time periods were obtained since samples for classical spectrophotometric measurements cannot be taken from the solidified compartmentalized aqueous inclusions.

**FIGURE 6:**
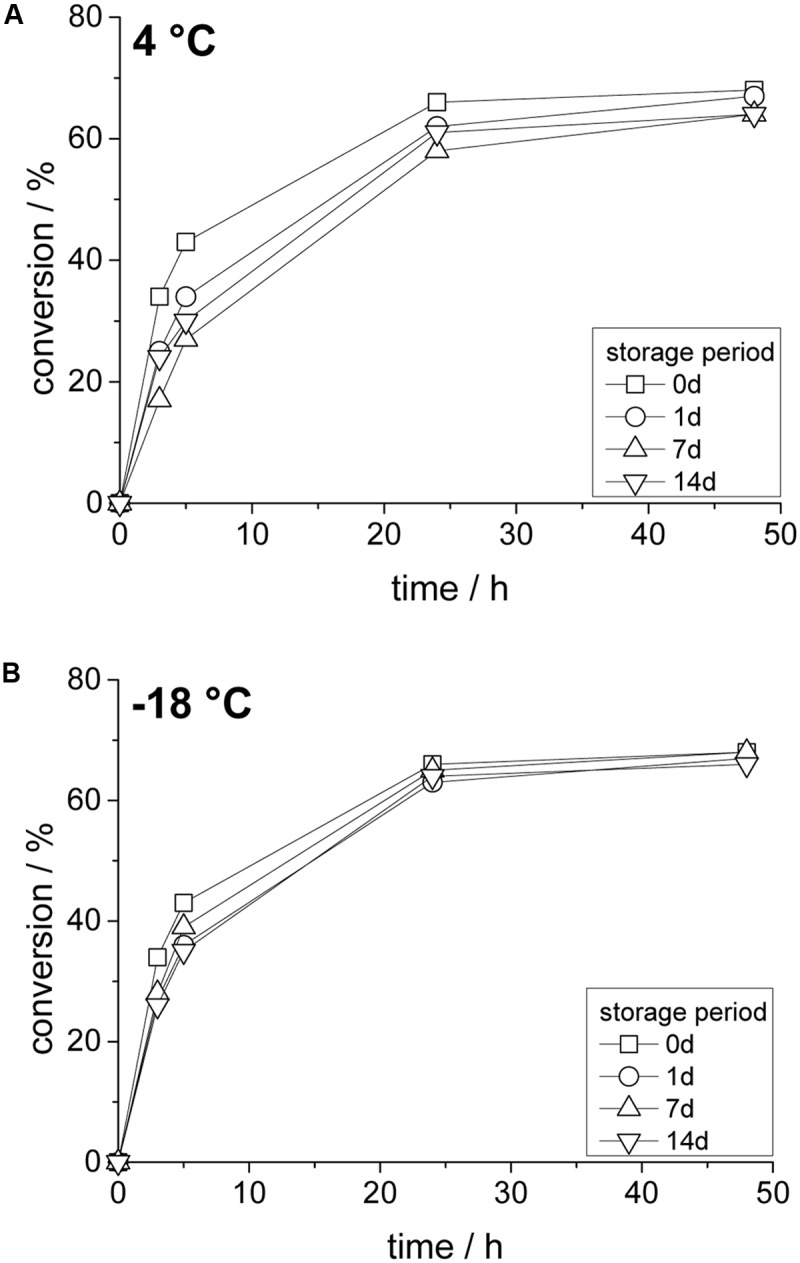
Effect of storage temperature and time on compartmented *Lk*ADH with substrate-coupled cofactor regeneration; **(A)** storage at 4°C, **(B)** storage at –18°C. Reaction conditions: 10 mM substrate in 5 mL MTBE, 100 mM 2-propanol (in external solvent).

As shown in **Figure 6A**, at 4°C only a small loss of enzyme activity was observed (<20% after 14 days), which is shown as a slightly lower initial reaction rate. However, sufficient enzyme activity is preserved and equilibrium conversion was also reached with 14 days old samples. Lower temperature improve enzyme stability even further and at -18°C only a marginal loss of enzyme activity was seen after 7 and 14 days of storage (**Figure 6B**). These results are consistent with stability measurements of a (classical) aqueous solution of *Lk*ADH at similar conditions, which highlights again the full compartmentalization of the entire aqueous phase. Unwanted secondary effects such as byproducts from curing or the negative side effect from the present hydrophobic surface were not observed.

### Model Cascade Reaction

**FIGURE 7:**
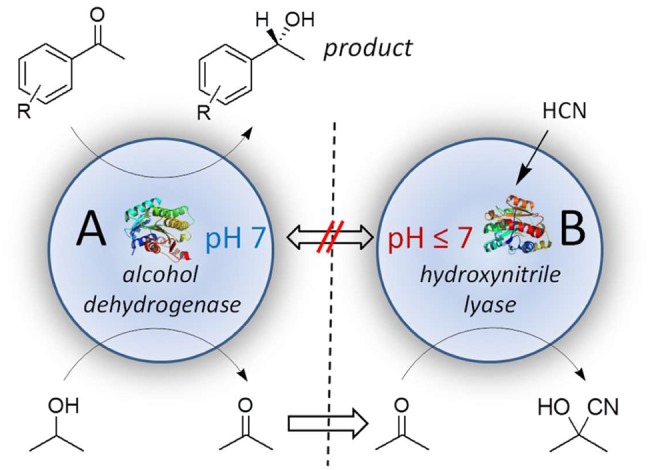
Compartmented cascade reaction with *Lk*ADH in compartment A at pH 7 and *Me*HNL in compartment B at pH < 7.

As a model reaction a combination of two very contrary reaction systems was chosen, the alcohol dehydrogenase from *Lactobacillus kefir*, *Lk*ADH (see also above) and hydroxynitrile lyase from *Manihot esculenta*, *Me*HNL (**Figure 7**). The shown example is intended as a model concept for biocatalytic systems, including alcohol dehydrogenases, with incompatible reaction requirements.

In reaction compartment A prochiral acetophenone is converted by *Lk*ADH to (*R*)-1-phenylethanol, which enriches in the external solvent phase. The oxidized cofactor NADP^+^ is simultaneously regenerated to NADPH + H^+^ by a substrate-coupled approach with 2-propanol yielding acetone (see also above). Acetone is then subsequently converted at a different pH to acetone cyanohydrin in reaction compartment B by *Me*HNL, representing principally an *in situ*-product removal (ISPR) of the co-product acetone.

**FIGURE 8:**
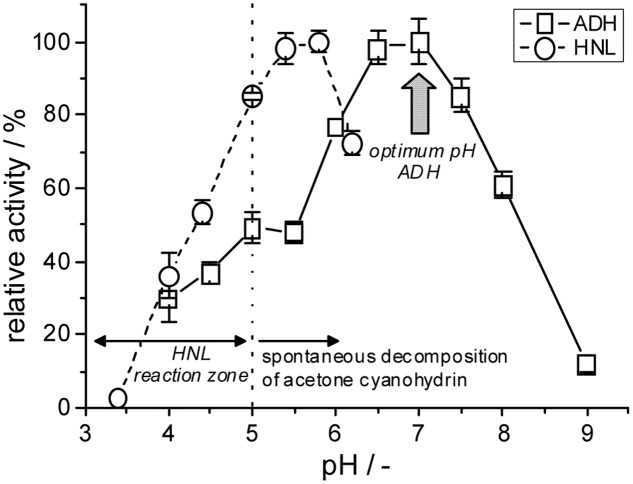
Comparison of the pH dependencies of the investigated alcohol dehydrogenase and hydroxynitrile lyase catalyzed reactions (solubilized, non-immobilized enzymes). Due to the low stability of acetone cyanohydrin the HNL-catalyzed reaction is in theory restricted to pH < 5 (Cooke, 1978).

The reaction requirements of these two biocatalysts are unfortunately significantly different. Alcohol dehydrogenase-catalyzed reductions of carbonyl compounds are typically performed at neutral pH-conditions and moderate reaction temperatures, e.g., 30°C. *Me*HNL on the other hand has its optimal reaction parameters at pH 5.5 and 60°C (Andexer et al., 2009; Guterl et al., 2009) (**Figure 8**). However, HNLs are generally used significantly below these parameters, e.g., in a pH range of 3–5 and temperatures below 10°C, which is required to suppress product racemization and decomposition of the respective cyanohydrin products (Von Langermann et al., 2008; Avi et al., 2009).

The shown compartmentation concept overcomes these problems by using two different polymer matrices each with specifically chosen optimal reaction parameters. *Lk*ADH was compartmented at pH 7, while the hydroxynitrile lyase remained at acidic conditions between pH 3 and 7. The relevant reactants, however, diffuse via the external solvent between the compartments (**Figure 7**) and are converted by the included biocatalysts. This reaction concept can be described as a multiphase-reaction system (external solvent, solid polymer phases, included aqueous domains in the compartments), which are interconnected by multiple diffusion systems.

Moreover, the chosen low water-content of polyurethane-based compartmentation is also relevant for the intended synthesis of acetone cyanohydrin (shown in **Figure 7**), which easily decompose in aqueous solutions, especially at pH > 5. Thus a hypothetical combination of both enzymes in an aqueous system is limited to pH < 5 and temperature of <10°C, which is not feasible for the applied alcohol dehydrogenase *Lk*ADH. Fortunately this is avoided by compartmentation, which significantly reduces these undesired side reactions by a full removal of overall water, except its minimum usage within the compartments for the enzyme (Von Langermann and Wapenhensch, 2014). Herein HNLs can now be used at optimal pH, higher temperatures and without loss of the desired cyanohydrin.

**FIGURE 9:**
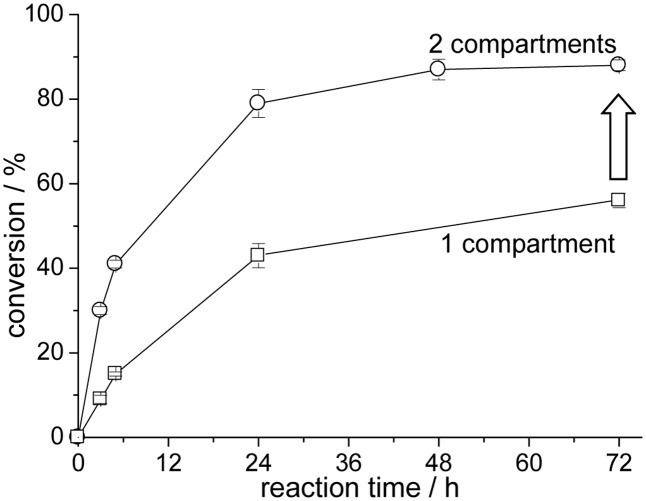
*Lk*ADH-*Me*HNL cascade reaction with one or two compartmented reaction zones; 1 compartment: only *Lk*ADH-compartments (open squares), 2 compartments: *Lk*ADH-compartments at pH 7 and *Me*HNL-compartments at pH 5 (open circles); reaction conditions: 30°C, 5 mL MTBE, 10 mM acetophenone, 100 mM 2-propanol and 200 mM hydrogen cyanide for the 2 compartment-reaction, enantiomeric excess of (*R*)-1-phenylethanol ≥ 99%.

As a result higher equilibrium conversions are obtained for the initial reduction reaction of acetophenone (**Figure 9**). The single compartmentation of the alcohol dehydrogenase-catalyzed (pH 7) reaction yields a conversion of only 56% after 72 h (filled triangle). The addition of the second hydroxynitrile lyase reaction compartment for the simultaneous synthesis of acetone cyanohydrin increased the conversion of the initial reduction reaction to 88%. This represents a significant improvement in comparison to the single compartmented enzyme reaction. In a side reaction acetophenone is also converted to (*S*)-acetophenone cyanohydrin by *Me*HNL in reaction compartment B, which fortunately only reduces the overall yield of the alcohol dehydrogenase-catalyzed reaction by a very small amount (von Langermann et al., 2007). In addition, similarly to a single compartmented *Lk*ADH-catalyzed reaction the ratio of prochiral ketone and 2-propanol, as part of the alcohol dehydrogenase-catalyzed reaction, affects the equilibrium conversion within the cascade reaction (Eckstein et al., 2006).

## Summary and Conclusion

In the presented work, the preparation and characterization of polyurethane-based materials for the compartmentation of enzymes and their subsequent use for biocatalytic reactions was investigated. The shown enzyme compartments were easily obtained by UV-light curing of an emulsion of a hydrophobic polyurethane precursor and an aqueous enzyme solution. Based on the formed initial emulsion the incorporated aqueous domains of the enzyme solution remain stable and finely dispersed in the polymer matrix, which itself is stable against various solvents.

As shown in this study, such a polymer-based compartmentation technique is compatible with alcohol dehydrogenase-, glucose dehydrogenase-, and hydroxynitrile lyase-catalyzed reaction systems. Negative effects for the included enzymes from the curing process or presence of the hydrophobic polymer phase (e.g., phase boundary within the polymer preparation) were not observed. The general synthetic applicability was shown for a single alcohol dehydrogenase-catalyzed reaction, enabling high conversions and enantiomeric excess of the respective chiral alcohols. In addition, different enzyme compartments with contrary reaction requirements can be used simultaneously in one reaction vessel, which facilitates the use of enzymatic cascade reaction in separated reaction zones with different reaction conditions. This includes also practically incompatible reaction conditions, as shown in the combined alcohol dehydrogenase-hydroxynitrile lyase-cascade reaction. Moreover, the shown polyurethane-based concept can be easily transferred to other (bio)catalytic reaction systems and further cascade reactions, especially with similar incompatible reaction requirements.

## Author Contributions

DU and JvL conceived and designed the study. DU performed the experiments. DU and JvL analyzed the data and prepared the manuscript.

## Conflict of Interest Statement

The authors declare that the research was conducted in the absence of any commercial or financial relationships that could be construed as a potential conflict of interest.
